# Not every nasal block is allergic rhinitis: a case report of sinonasal malignant melanoma

**DOI:** 10.1093/jscr/rjaf295

**Published:** 2025-05-13

**Authors:** Rula Naqi, Vineeth Haridas, Ismail Kandiyil, Mohammed Al-Shehabi, Mai Nasser, Amal Al-Abdullah

**Affiliations:** Otolaryngology Department, Bahrain Defence Force Hospital- Royal Medical Services, Rd No 2802, PO Box 28743, Riffa, Kingdom of Bahrain; Otolaryngology Department, Bahrain Defence Force Hospital- Royal Medical Services, Rd No 2802, PO Box 28743, Riffa, Kingdom of Bahrain; Otolaryngology Department, Bahrain Defence Force Hospital- Royal Medical Services, Rd No 2802, PO Box 28743, Riffa, Kingdom of Bahrain; Otolaryngology Department, Bahrain Defence Force Hospital- Royal Medical Services, Rd No 2802, PO Box 28743, Riffa, Kingdom of Bahrain; Otolaryngology Department, Bahrain Defence Force Hospital- Royal Medical Services, Rd No 2802, PO Box 28743, Riffa, Kingdom of Bahrain; Otolaryngology Department, Bahrain Defence Force Hospital- Royal Medical Services, Rd No 2802, PO Box 28743, Riffa, Kingdom of Bahrain

**Keywords:** sinonasal tumor, malignant melanoma, oncology, treatment guidelines, rhinology

## Abstract

Sinonasal malignant melanoma (SNMM) is an extremely rare and aggressive sinonasal tumor. It has nonspecific risk factors and clinical presentation which require a high index of suspicion. In this case report we present a case of a 47-year-old male who initially had persistent nasal obstruction not responding to intranasal steroids. Comprehensive imaging and histopathological studies confirmed SNMM. He underwent incomplete maxillectomy resection followed by immunotherapy abroad. Within 6 months period he had recurrence of the disease. As this disease is a rare entity, there are no specific treatment guidelines, however, surgical intervention is agreed to be the mainstay of treatment. Although their effectiveness is still controversial, radiation therapy, chemotherapy, immunological and biological therapies are adjuvant options. Multidisciplinary team approach is necessary to provide the optimal treatment plan, along with close follow up. Continuous research and clinical trials are still needed to ensure appropriate diagnosis and management of this rare entity.

## Introduction

Melanocytes are dendritic cells containing melanin arising from neural tube [1]. Gilain *et al.* identified the presence of these cells in the nasal cavity [1]. Sinonasal malignant melanoma (SNMM) arise from these cells and represent 0.5%–1% of all otolaryngology tumors [1]. The absence of clear risk factors and slow development of non-specific symptoms delay the diagnosis [1]. Moreover, dilemma exists regarding the optimal treatments, which include surgical resection, radiotherapy, chemotherapy, and biological therapy [1]. We herein report the first case of SNMM in our region, up to our knowledge, and we elaborate on the clinical presentation, treatment, and outcomes.

## Case report

A 47-year-old male was referred to the otolaryngology clinic with history of left nasal block, rhinorrhea and suspected vestibulitis of 1 month duration. It did not improve with intranasal steroids. Upon nasal flexible scope examination, there was a left sided fleshy mass markedly affecting the airway. Computed tomography (CT) of the paranasal sinuses with contrast was requested.

**Figure 1:**
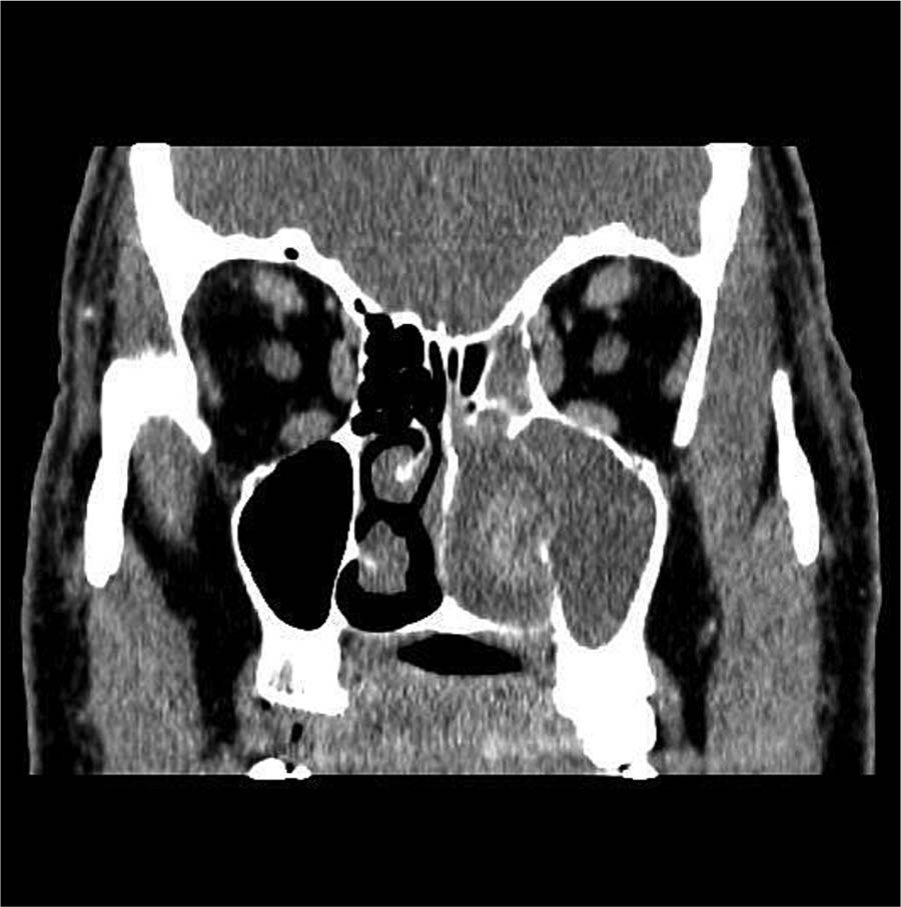
Coronal section of CT paranasal sinus with contrast soft tissue window showing the mass occupying the left nasal cavity and extending to maxillary and ethmoidal sinus.

**Figure 2:**
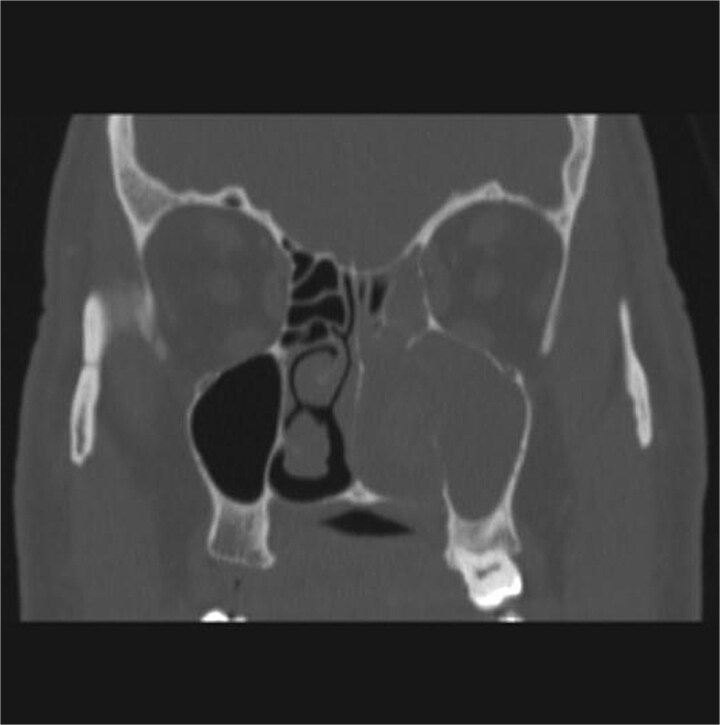
Coronal section of CT paranasal sinus with contrast bone window showing the bony erosion in the nasal septum and maxillary sinus wall.

CT showed a heterogeneously enhancing mass completely occluding the left nasal cavity, left ethmoidal, maxillary and frontal sinuses, extending to left pterygopalatine fossa. It is associated with bony erosion of the posterior nasal septum and the left medial maxillary wall (Figs 1 and 2). Findings were suggestive of inverted papilloma vs sinonasal malignancy. Therefore, biopsy was recommended.

Histopathology showed a necrotic tumor of epithelioid and spindle cells, having eosinophilic cytoplasm with prominent nucleoli, occasional mitosis and apoptosis. Immunohistochemistry was positive for CD56, CD99, S100, HMB45, Melan-A, and Ki67. This confirmed the diagnosis of malignant melanoma.

**Figure 3:**
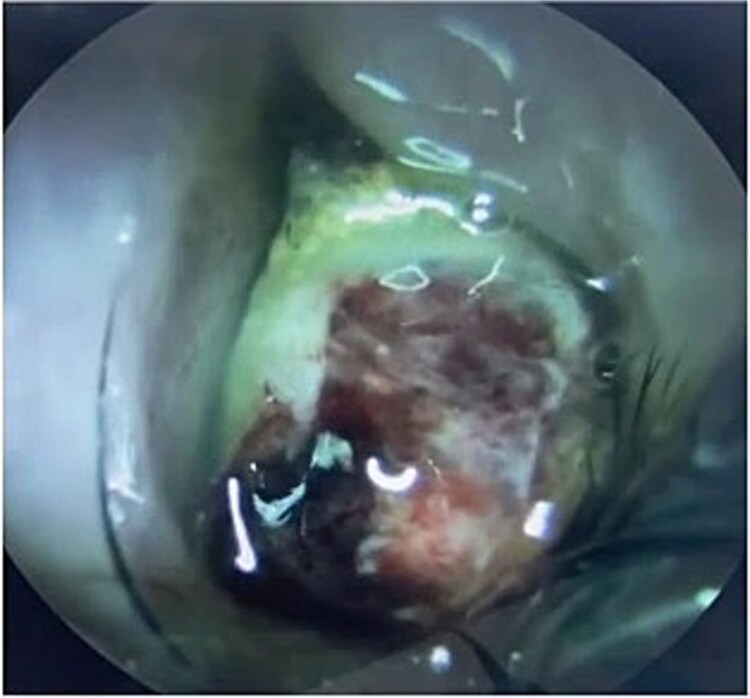
Yellowish greenish nasal mass occupying the left nasal cavity with blood stain.

**Figure 4:**
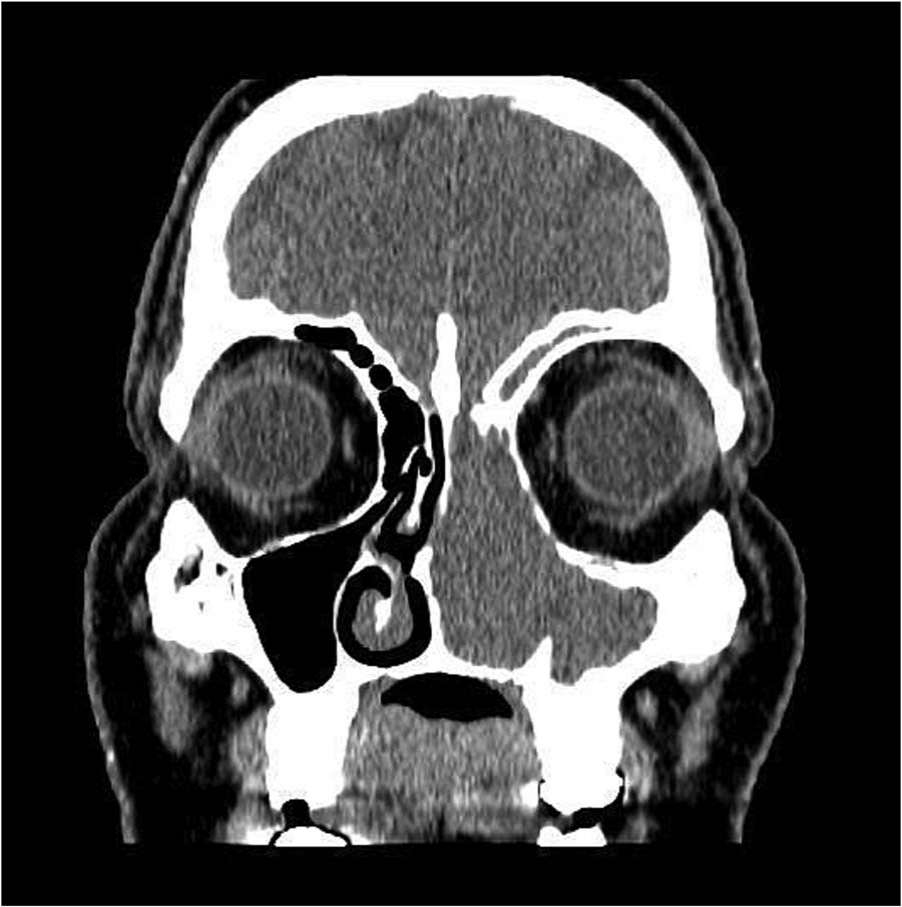
Coronal section of CT paranasal sinus soft tissue window showing the mass occupying the left nasal cavity and extending to maxillary and ethmoidal sinus, and reaching base of skull.

**Figure 5:**
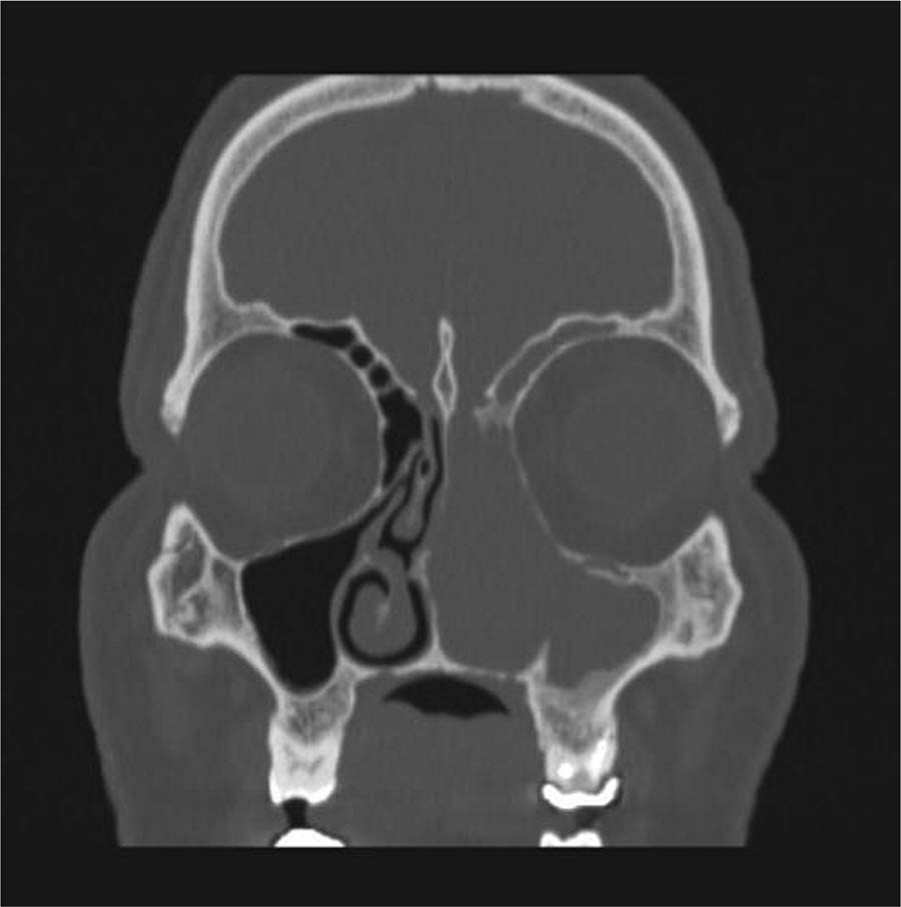
Coronal section of CT paranasal sinus bone window showing bony erosions of nasal septum, maxillary wall, lamina papyracea, and cribriform plate.

The patient underwent staging CT which was negative for distant metastasis. He then went underwent incomplete medial maxillectomy, ethmoidectomy, and sphenoidotomy followed by 10 sessions of immunotherapy abroad. He presented to our institution for follow up 6 months later with history of left nasal block and epistaxis. Nasal flexible scope showed a reddish nasal mass with greenish secretions (Fig. 3). Follow up CT revealed tumor recurrence (Figs 4 and 5). He was advised for multidisciplinary team evaluation to plan the treatment but he refused any intervention and lost follow up.

## Discussion

### Definition and epidemiology of SNMM

SNMM is a rare type of cancer that arises from melanocytes within the sinonasal cavity mucosa, predominantly along the septum, lateral wall of nasal cavity and maxillary sinus [1]. The prevalence of mucosal malignant melanoma is 1.3% with 40%–55% of them seen in the head and neck [2]. In our case the tumor involved the nasal septum, maxillary and frontal sinuses as well as the ethmoidal air cells.

### Pathogenesis of SNMM

Melanocytes are dendritic cells found in dermoepidermal junction of all mucous membranes and neural tube [1]. Melanocytes usually tend to appear in the sinonasal cavity under the age of 19 years and are detected in around 21% of individuals [3]. Melanocytes density in sinonasal cavity is higher than other mucosal sites which explain the tumor frequency [3]. There are no clear risk factors, however, studies showed association with smoking and formaldehyde [1]. Moreover, genetic studies showed tyrosine kinase receptor gene mutations involved [4].

### Clinical presentation of SNMM

Patients tend to be asymptomatic, but the commonest presentations are unilateral nasal obstruction and epistaxis [1]. Moreover, rhinorrhea, lacrimation, nasal deformity, and exophthalmos can be presenting complains [5]. Non-specific symptoms could delay the diagnosis; therefore, clinicians should have a high clinical index of suspicion. In our case, the diagnosis was delayed as it was treated initially as a case of allergic rhinitis. Physical examination by endoscopy is essential to assess the tumor features. It could be nodular, polypoidal, or ulcerated, with variable colors but sometimes achromic [1]. Moreover, cervical lymph nodes examination and comprehensive dermatology evaluation are essential. Distant metastasis to the lungs, brain, bone and liver should be excluded.

### Investigations of SNMM imaging

Imaging is crucial to identity the extent of the disease and plan treatment. CT scans provides clear outline of the tumor, facial bones and skull base [1]. Magnetic resonance imaging yields high-resolution images that show high signal intensity on T1 and low signal on T2 which are typical of melanoma [6]. CT chest, abdominal, and pelvic CT and positron emission tomography scans should be utilized to exclude any distant metastasis [1, 6].

### Histology

The final diagnosis of SNMM is based on immunohistochemistry as histopathological diagnosis can lead to diagnostic errors [1]. Histopathological differential diagnoses include sinonasal undifferentiated carcinoma, lymphoma, rhabdomyosarcoma, angiosarcoma, and neuroendocrine carcinoma [1]. SNMM often appear as large fusiform or epithelioid cells with abundant eosinophilic cytoplasm, or they may be amelanotic. S-100 protein, HMB-45, melan-A, microphthalmia-associated transcription factor, tyrosinase, vimentin, and cytokeratin immunohistochemical markers can be positive [6].

### Treatment of SNMM

The treatment for SNMM should focus on optimizing the quality of life of the patient as they may have poor prognosis [1]. The choice of optimal treatment necessitates a multidisciplinary team approach [1, 7].. The cornerstone of treatment is complete surgical resection, which improves prognosis, yet, positive margins result in higher rates of distant metastasis [7, 8]. The role of radiotherapy in SNMM is controversial, as the tumor has low radiosensitivity, and is generally indicated for positive surgical margins, local recurrence, or advanced disease [1]. Immunotherapy is a debatable treatment option [9]. In our case, the patient received 10 cycles of adjuvant immunotherapy, however, he had disease recurrence. Moreover, chemotherapy is usually given as an adjuvant and can be used in cases with metastasis or failure of surgical treatment [7]. Dacarbazine is the most commonly used chemotherapeutic agent [1]. Furthermore, trial of biological therapy have showed good response and tolerability with MEK and BRAF [10].

### Prognosis and recurrence

The survival rates for sinonasal malignant melanoma vary, with a reported 5-year survival of 30.9% for tumors of the nasal cavity and 0% for those of the sinuses [1]. The presence of a multifocal tumor and local implantation during surgery can be contributing factors [8]. In our case, the patient had local recurrence six month post-operatively. Residual disease might have been present after the incomplete medial maxillectomy or local implantation during surgery which could explain the recurrence. Hence, we emphasize the necessity of complete resection with clear margins by surgical intervention.
